# Traumatic spinal cord injury and its correlation to risk of autoimmune/-inflammatory disease

**DOI:** 10.1038/s41393-024-01026-0

**Published:** 2024-09-11

**Authors:** Tim Damgaard Nielsen, Thomas Munk Laursen, Bodil Hammer Bech, Mikkel Mylius Rasmussen

**Affiliations:** 1 Cense Spine, Department of Neurosurgery, Aarhus University Hospital, Aarhus N, Denmark; 2 Department of Clinical medicine, Aarhus University, Aarhus N, Denmark; 3 Department of Economics and Business economics, Aarhus University, Aarhus V, Denmark; 4 Department of Public Health, Aarhus University, aarhus, Denmark; 5 Department of Epidemiology, Aarhus University, Aarhus, Denmark

**Keywords:** Risk factors, Epidemiology, Neurology

## Abstract

**Study design:**

Nationwide epidemiological open cohort study.

**Objectives:**

To evaluate whether individuals with traumatic spinal cord injury (TSCI) are more prone to develop autoimmune diseases compared to a general non-TSCI population.

**Setting:**

Danish public national registries.

**Methods:**

An open nationwide cohort, including individuals born in Denmark from or alive during 1945-2018 was collected and the study period was 1980-2018. Poissons Log-linear regression estimated the incidence rate ratio (IRR) for developing eight groups of autoimmune diseases. A dose-response relationship based on the cervical/thoracic level of injury was assessed by stratification.

**Results:**

The cohort included 3,272 individuals with TSCI and 4.8 million background individuals, accounting for 50,865 and 140 million person-years respectively. The TSCI population had an overall IRR of 1.81 (95% CI, 1.59 to 2.05) of getting any autoimmune disease. Subgroup analysis found positive associations for; a) *Other neurologic* IRR 5.19 (95% CI, 2.79 to 9.65), b) *multiple sclerosis* IRR 3.70 (95% CI, 2.54 to 5.40), c) *Dermatologic* IRR 2.57 (95% CI, 1.86 to 3.55), d) Type 1 d*iabetes mellitus* IRR 2.01 (95% CI, 1.54 to 2.61), e) *Systemic* 1.92 (95% CI, 1.44 to 2.55), and f) *Gastroenterologic* IRR 1.42 (95% CI, 1.05 to 1.92). Cervical levels of TSCI showed an IRR of 1.70 (95% CI, 1.43 to 2.02), while thoracic levels had an IRR 1.98 (95% CI, 1.63 to 2.39).

**Conclusions:**

TSCI may be an individual risk factor of developing an autoimmune disease. There does not appear to exist a dose-response relationship from the level of injury.

**Sponsorship:**

None.

## Introduction

The worldwide incidence of traumatic spinal cord injury (TSCI) ranges from 5.1 – 150.48 per million person-years [1]. The neurologic injury affects motor-, and sensory functions, bladder, bowel, and sexual function, and TSCI could be seen as a multiorgan disease [2]. Experimental-, clinical-, and observational studies suggest a deteriorating immunological response following TSCI, which may lead to secondary neurologic injury, multiorgan disease and autoimmune disease ultimately increasing morbidity and mortality [2–4].

Trauma induces the primary neurological injury. Concomitant cellular damage and the presumably compromised blood spinal cord barrier trigger a deteriorating inflammatory cascade [4, 5]. Myeloid cells’ phagocytosis of myelin turns them into foamy cells resulting in apoptosis/necrosis further fueling the inflammatory process [5]. Eventually, autoreactive T- and B-cells are activated, myelin-specific autoantibodies can be measured, and chronic inflammation is established [4, 6–8].

The high inflammatory activity initiates spinal cord injury immunodeficiency syndrome (SCI-IDS), a state of systemic immunosuppression, causing secondary neurologic injury with prolonged self-epitope exposure between antigen-presenting and endemic autoreactive lymphoid cells [4, 9]. The ongoing inflammation and secondary neurologic injury prolong exposure to central nervous system self-epitopes increasing susceptibility to match antigen-presenting cells with autoreactive lymphoid cells; a door that enables autoimmune development [4, 7, 9, 10].

The immunosuppressive state of SCI-IDS may have a protective effect against autoimmune development [11]. However, the same effect also increases susceptibility to infection, which leads to higher rates and severity of hospitalizations for infectious diseases. Post-TSCI infectious diseases are known to affect neurological recovery negatively. Hospitalization for an infectious diseases is a known risk factor of developing any autoimmune disease [9, 11, 12].

Evidence suggest that more cranial injuries amplify both SCI-IDS and autonomic dysreflexia [3, 11, 13]. Autonomic dysreflexia is immunosuppressive due to the high-level releases of norepinephrine and glucocorticoids during attacks [3]. The balance of beneficial and detrimental effects of SCI-IDS and autonomic dysreflexia is likely level-dependent, and the risk of post-TSCI autoimmunity may also be level-dependent [9].

Trauma presumably tears the blood-spinal cord barrier and discontinues the immune-privilege of the central nervous system [14]. This allows unregulated access of the systemic immune system and unphysiological drainage of central nervous system debris to secondary lymphoid tissue [10]. Research into central nervous system drainage pathways and the immune privilege is ongoing, but susceptibility to autoimmunity could be feasible due to the physiological/immunological changes from TSCI, combined with the fact that central nervous system cells have a lower expression of major histocompatibility complex-I and -II receptors [15]. Under normal physiological circumstances, immune cells are presented with fewer central nervous system autoantigens, possibly lowering tolerance to self compared to systemic antigens [5–7, 15]. This, along with prolonged self-epitope exposure, could explain why TSCI might lead to posttraumatic immunological dysfunction, resulting in autoimmunity/-inflammation, multiorgan, and autoimmune diseases, and higher posttraumatic morbidity and mortality [5, 7, 8, 13, 16].

The aetiology to autoimmune disease is heavily multifactorial. Some general risk factors have been identified for autoimmune disease including genetics, prior autoimmune disease, and hospitalization for an infection which significantly increase the risk of any autoimmune disease [12, 17–19]. However, some studies suggest that specific infections might be protective against some autoimmune diseases [20, 21].

Whether these findings and theories have clinical relevance for the long-term morbidity of TSCI individuals is unknown. There is an important distinction between the evident sensitization to self and the non-investigated symptomatic disease. Based on previous findings, we hypothesized that individuals with TSCIs would be at a higher risk of developing an autoimmune disease following trauma. We aimed to identify if an association exists between TSCI and diagnosed autoimmune disease.

## Method

### Study design, -population, and -period

**Fig. 1:**
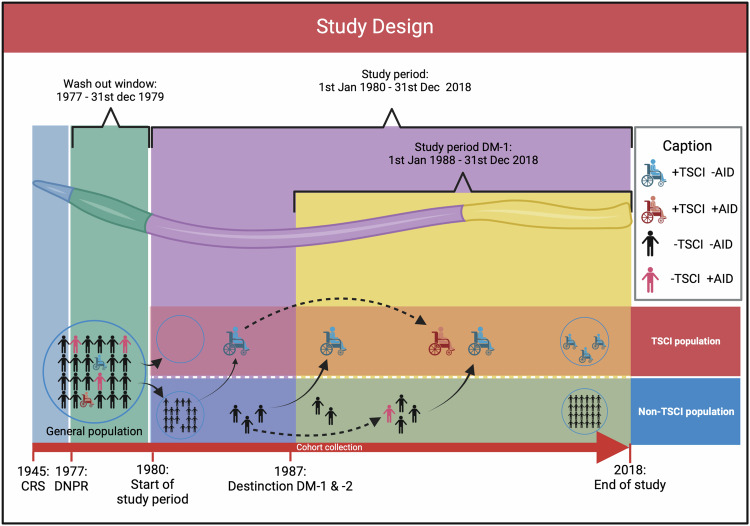
Display of the study design. From 1945–1976 (blue period) people born in Denmark, who are still alive are registered. Registration continues until the end of the study. From 1977–31st Dec 1979 (green period) prevalent cases with either TSCI or AD are excluded from the study, leaving the TSCI population empty at the start of the study period. The purple period marks the main study period, where TSCIs are collected and risk time is counted until any endpoint is reached and here from censoring from the study. The yellow period marks the study period for DM-1. CRS Civil Registration System, DNPR Danish National Patient Registry, DM-1 Diabetes Mellitus type 1, DM-2 Diabetes Mellitus type 2, TSCI Traumatic Spinal Cord Injury, AID Autoimmune Disease.

A nationwide cohort of individuals born in Denmark between 1st January 1945, and 31st December 2018 was identified. Individuals had to be registered alive and residing in Denmark in the Danish Civil Registration System during the study period from 1st January, 1980–1st December, 2018. The years 1977–1979 were used as a washout to exclude prevalent cases of autoimmune disease and TSCI (see Fig. 1). Individuals (of any age; including pediatric) were longitudinally tracked from birth or 1st January 1980, whichever occurred later, until they reached an endpoint. Endpoints included the diagnose of autoimmune disease, death, emigration from Denmark, diagnosis of a spinal tumor, or the conclusion of the study on 31st December, 2018, whichever came first.

Specifically for Type 1 diabetes mellitus (DM-1), this condition was first possible to distinguish from other types of diabetes in the Danish national patient registry from 1987 and onwards. The design for this outcome was similar, with 1987 serving as the washout period for prevalent cases, and the study period for DM-1 spanning from 1988–2018 (see Fig. 1).

### Study variables

TSCI was identified from the Danish national patient registry. We utilized ICD-8 and ICD-10 codes to identify nine diagnoses covering cervical and thoracic types and levels of TSCI (see Supplementary 1 and Section 2.3). Paraplegia/tetraplegia and cord compression (ICD-8: 343.02, 343.03, and ICD-10: G82 and G952) had to be registered within +/- 7 days of a spinal fracture diagnosis and not in relation to a cancer diagnosis -10 year/+7 days timeframe. Conus medullaris TSCI (ICD-8: 958.11, 958.12, 958.13, 958.14, 958.18, 958.19 and ICD-10: S340, S341B, -C, -D, -E) was excluded, as its incidence was deemed inconsistent with clinical reality. ICD-8 diagnosis codes of 805.23, 805.31, 805.33, 805.69, and 805.79 covering different types of spinal fracture were not registered in during the study period and therefore removed from the statistical analysis.

In a secondary analysis, we categorized TSCI as either cervical or thoracic level of injury to evaluate a possible dose-response relationship based on the region of injury. The categorization is detailed in Supplementary 1.

As primary outcome was 31 autoimmune/-inflammatory diseases, categorized into eight groups as listed in Supplementary 2. Grouping was done to maintain confidentiality. The time of diagnosis was set to the first hospital contact for medical elucidation of autoimmune/-inflammatory disease ending with a diagnosis of such. For the TSCI group, an autoimmune disease diagnosis was further restricted as follows: a diagnosis of TSCI and autoimmune disease during the same hospital stay resulted in the exclusion of both TSCI and autoimmune disease and the individual was censored from the study. Supplementary 2 lists the autoimmune diseases and their grouping and Fig. 1 displays the study period for the various outcomes.

### Registries

Statistics Denmark maintains the public Danish registries including The Civil registration system and Danish national patient registry. Since 1968, the Civil registration system has recorded information on people residing in Denmark. From here we have recorded; birthday, birth country, sex, migration status, death, and the person-specific and unique Civil registration system number [22]. Sex is identifiable from the Civil registration system number [22]. The Danish national patient registry contains information on all in- and outpatients visits, including diagnosis and temporal context. These variables were included in the study. Registration began in 1977 using the ICD-8 system, and the Danish national patient registry transitioned to the ICD-10 in 1994. Discrimination between type 1 and type 2 diabetes started in 1987 (see Fig. 1) [23, 24].

### Co-variates

**Fig. 2:**
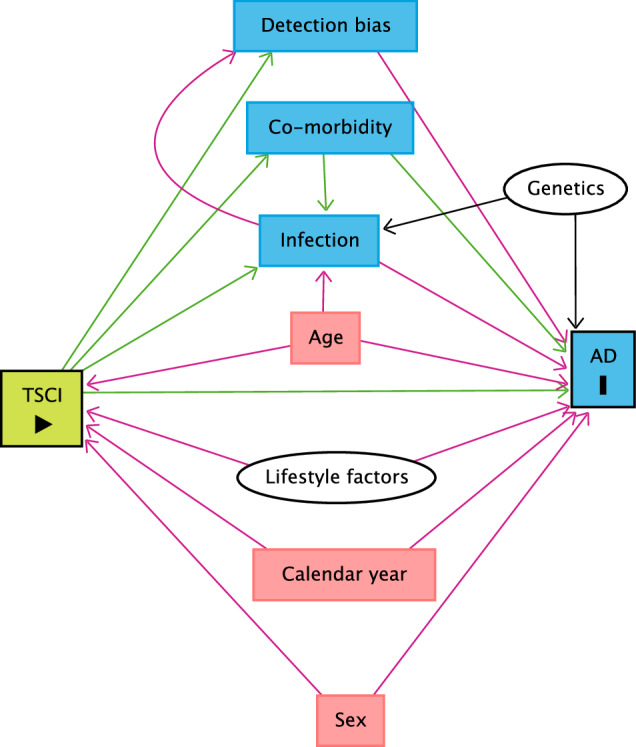
Directed Acyclic Graph showing TSCI as exposure, and AD as outcome. Six mediated paths going; 1: TSCI → Detection bias → AID. 2: TSCI → Co-morbidity → AID. 3: TSCI → Co-morbidity → Infection → AID. 4: TSCI → Co-morbidity → infection → Detection bias → AID. 5: TSCI → Infection → AID6: TSCI → Infection → Detection bias → AID. In red is marked confounding paths. Genetics and lifestyle factors (marked with white) are unobserved and cannot be adjusted or restricted in the analysis. TSCI Traumatic spinal cord injury, AID Autoimmune disease.

Charlson Comorbidity index was included in the analysis as a mediator to adjust for comorbidities and highlight the burden among individuals with TSCIs. The validated 2011 version from Christensen et. al. was used [25]. Charlson comorbidity index was evaluated as a time-dependent variable and modified as described in Supplementary 3, with the exclusion of connective tissue disease (an outcome), viral liver disease (included in hospitalization for an infection variable), primary biliary cirrhosis (an outcome), diabetes type 1 and 2, para- and tetraplegia (exposure), and AIDS. Diabetes type 1 was excluded as it is an outcome. Diabetes type 2 was excluded because most cases are managed by general practitioners who do not report diagnosis to the included registries. Therefore, diabetes type 2 was only adjusted based on diabetes complications that are managed in-hospital and reported to the included registries. AIDS was excluded as it was not considered a confounder. Age, sex, and calendar year were considered confounders. Hospitalization for infection was stratified in bacterial, viral, and other infections, evaluated as mediators with categorical variables as either 0 = *no*, 1 ≤ *5 years since hospitalization for infection*, or 2 ≥ *5 years since hospitalization for infection*, according to the study by Nielsen P.R. et al. [12] (see Table 1). Interpretation of the causal diagram is shown in Fig. 2.

**Table 1:** Baseline characteristics.

	Demographics of study population by traumatic spinal cord injury status					
	TSCI population			Non-TSCI population		
	PY at risk (%)	Outcomes (%)	Rate; 1000^-1^	PY at risk^A^ (%)	Outcomes (%)	Rate; 1000^-1^
**Age**						
0–19 y	3341 (6.6)	5 (2.1)	1.5	0.48 (35)	40,887 (15)	0.85
20–39 y	22,730 (44.7)	76 (32)	3.3	0.51 (37)	98,852 (36)	1.9
40–59 y	21,036 (41.4)	120 (51)	5.7	0.33 (24)	105,830 (39)	3.2
60–y	3758 (7.4)	35 (15)	9.3	0.06 (4.3)	28,745 (11)	4.8
**Mean risk time PY**		15.6			28.32	
**Autoimmune diseases (n)**		236			274,314	
**Sex**						
Female	14,070 (27.6)	71 (30)	5.0	0.68 (49)	156,999 (57)	2.3
Male	36,795 (72.4)	165 (70)	4.5	0.71 (51)	117,315 (43)	1.7
**Calendar year**						
1980–1989	4481 (8.8)	7 (3.0)	1.6	0.28 (21)	20,854 (7.6)	0.73
1990–1999	10,977 (21.6)	37 (14)	2.9	0.34 (24)	55,473 (20)	1.6
2000–2009	16,414 (32.3)	84 (36)	5.1	0.38 (28)	95,192 (35)	2.5
2010–2018	18,992 (37.3)	113 (48)	6.0	0.37 (27)	102,795 (38)	2.7
**mCCI groups**						
0	39,412 (77.5)	156 (66)	4.0	1.3 (94)	225,725 (82)	1.7
1	4689 (9.2)	37 (16)	7.9	0.06 (4.2)	26,152 (9.5)	4.4
2	4976 (9.8)	23 (9.7)	4.6	0.02 (1.6)	13,567 (4.9)	6.1
≥3	1788 (3.5)	20 (8.5)	11.2	0.01 (0.5)	8870 (3.2)	12
**Hospitalization for bacterial infection**						
No	31,227 (61.4)	101 (43)	3.3	1.2 (88)	204,989 (75)	1.7
<5 y	5279 (10.4)	39 (17)	7.4	0.05 (3.6)	24,580 (9.0)	5.0
≥5 y	14,359 (28.2)	96 (41)	6.7	0.12 (8.4)	44,745 (16)	3.9
**Hospitalization for viral infection**						
No	46,120 (90.7)	210 (89)	4.6	1.3 (93)	248,823 (91)	1.9
<5 y	833 (1.6)	7 (3.0)	8.4	0.03 (1.9)	7540 (2.7)	2.9
≥5 y	3912 (7.7)	19 (8.1)	4.9	0.07 (5.0)	17,951 (6.5)	2.6
**Hospitalization for other infection**						
No	35,250 (67.6)	132 (56)	3.7	1.2 (84)	206,533 (75)	1.8
<5 y	2643 (5.5)	26 (11)	9.8	0.05 (3.8)	18,836 (6.9)	3.6
≥5 y	12,971 (26.9)	78 (33)	6.0	0.16 (12)	48,945 (18)	3.0
**Mean age for TSCI population at time of injury (SD)**						
1980–1989		25 (7.7)			..	
1990–1999		28 (11)			..	
2000–2009		33 (16)			..	
2010–2018		38 (19)			..	

*TSCI* Traumatic spinal cord injury, *PY* Person years, *y* years, *mCCI* modified Charlson Comorbidity Index, *n* counts, *SD* Standard deviation.

A: All person years are in 10^8^ exponent.

### Statistical analysis

The statistical analysis was conducted as a survival analysis using data recorded from 1st January, 1980–31st December, 2018, employing Poisson Regression with person years at risk as an offset variable. This model is equivalent to a Cox regression model [26, 27]. We used the Genmod procedure in the statistical software SAS version 9.4 (SAS Institute Inc., USA). Risk time was calculated from entry to the study until the individual reached the first endpoint, at which point they were censored from the study. An individual’s risk time was included in all outcomes, but only an individual’s first diagnosed autoimmune disease counted if that was the first temporal endpoint. We constructed three adjusted analyses, incorporating covariates from the previous model; (1) Basic adjustment: Included sex, age (in one‐year groups), and calendar year (in one‐year groups). (2) Charlson comorbidity index adjustment; Further adjusted for Charlson comorbidity index as time-dependent covariate. (3) Fully adjusted; Further adjusted for hospitalization for bacterial, viral, and other) infections (see Supplementary 4).

## Results

The study included 4,874,564 individuals, accounting for 138,046,152 risk years. Of these 3272 was diagnosed with TSCI, accumulating 50,865 risk-years. The median annual TSCI incidence was 72 (IQR 64.5; 80.0). The annual incidence of TSCI is displayed in Supplementary 5.

Basic demographics of the TSCI and non-TSCI populations are displayed in Table 1.

**Fig. 3:**
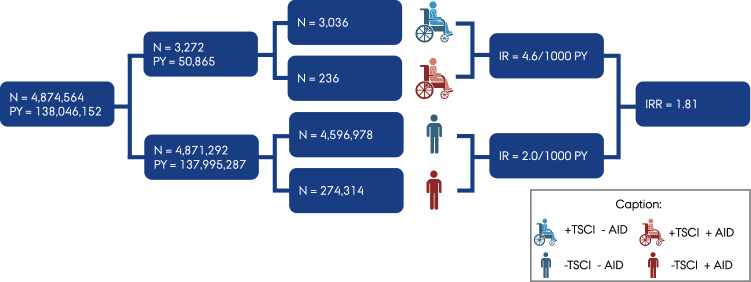
Graphical abstract of main outcome. Displaying a selection of study participants and accumulated person-years and the main outcome of the study; an overall increased IRR of 1.81 of being diagnosed with an autoimmune disease given a preexisting TSCI compared to the Danish background population.N counts, PY Person years, IR Incidence rate, IRR Incidence rate ratio AID Autoimmune Disease.

In total, 236 individuals with TSCI and 274,314 without TSCI had an autoimmune disease diagnosis (see Fig. 3). The incidence rate ratios (IRR) of being diagnosed with an autoimmune disease given a pre-existing TSCI are shown in Table 2 with three models displayed: From basic adjusted to fully adjusted model. TSCI showed either an increased IRR of being diagnosed with an autoimmune disease or no association across all outcomes. The overall incident association was IRR 1.81 (95% CI, 1.59 to 2.05) and IRR 1.32 (95% CI, 1.16 to 1.50) in the basic- and fully adjusted models, respectively. Among the eight groups of autoimmune diseases, the strongest association in the basic adjusted model were (a) *Other neurologic* IRR 5.19 (95% CI, 2.79 to 9.65), including myasthenia gravis and idiopathic polyneuropathy, (b) *Multiple sclerosis* IRR 3.70 (95% CI, 2.54 to 5.40), (c) *Dermatologic* IRR 2.57 (95% CI, 1.86 to 3.55), (d) *DM-1* IRR 2.01 (95% CI, 1.54 to 2.61), (e) *Systemic* IRR 1.92 (95% CI, 1.44 to 2.55), and *Gastroenterologic* IRR 1.42 (95% CI, 1.05 to 1.92). *Endocrine & haematologic* and *Iridocyclitis* autoimmune diseases showed no association in the basic adjusted model. All associations were uniformly mitigated through adjustment. The significant associations for *Gastroenterologic* and *DM-1* were eliminated in the Charlson comorbidity index- and fully adjusted models, respectively. The All-incident analysis is in supplementary 6 grouped in TSCI diagnosis before or from 1995 and forward as means of sensitivity analysis of TSCI diagnosis from ICD-8 and ICD-10 respectively. There was no significant difference.

**Table 2:** Study outcomes.

	Incidence rate ratios of autoimmune disease associated with traumatic spinal cord injury						
					1: Basic adjustment^A^	2: CCI adjusted^B^	Fully adjusted^C^
Auoimmune disease group	Exposure status	N	PY^D^	Rate; 1000^−1^		Incidence rate ratio (95% CI)	
**All incident types**	TSCI	236	50,865	4.6	1.81 (1.59 to 2.05)	1.53 (1.35 to 1.74)	1.32 (1.16 to 1.50)
	Non-TSCI	274,314	1.4	2.0	1.00 reference	1.00 reference	1.00 reference
**Endocrine & haematologic**	TSCI	14	50,865	0.28	0.88 (0.52 to 1.48)	0.79 (0.47 to 1.33)	0.72 (0.43 to 1.22)
	Non-TSCI	39,125	1.4	0.28	1.00 reference	1.00 reference	1.00 reference
**Gastroenterologic**	TSCI	42	50,865	0.83	1.42 (1.05 to 1.92)	1.22 (0.90 to 1.66)	1.06 (0.78 to 1.43)
	Non-TSCI	65,220	1.4	0.47	1.00 reference	1.00 reference	1.00 reference
**DM-1**^E^	TSCI	55	47,803	1.2	2.01 (1.54 to 2.61)	1.39 (1.07 to 1.81)	1.13 (0.87 to 1.47)
	Non-TSCI	51,846	1.2	0.45	1.00 reference	1.00 reference	1.00 reference
**Iridocyclitis**	TSCI	7	50,865	0.14	0.86 (0.41 to 1.80)	0.80 (0.38 to 1.69)	0.70 (0.34 to 1.48)
	Non-TSCI	14,449	1.4	0.11	1.00 reference	1.00 reference	1.00 reference
**MS**^F^	TSCI	27	59,836	0.54	3.70 (2.54 to 5.40)	3.46 (2.37 to 5.04)	3.23 (2.21 to 4.71)
	Non-TSCI	15,709	1.1	0.15	1.00 reference	1.00 reference	1.00 reference
**Dermatologic**	TSCI	37	50,865	0.73	2.57 (1.86 to 3.55)	2.26 (1.63 to 3.11)	1.90 (1.37 to 2.62)
	Non-TSCI	28,164	1.4	0.20	1.00 reference	1.00 reference	1.00 reference
**Other neurologic**	TSCI	10	50,865	0.20	5.19 (2.79 to 9.65)	4.37 (2.35 to 8.14)	3.71 (1.99 to 6.91)
	Non-TSCI	4273	1.4	0.03	1.00 reference	1.00 reference	1.00 reference
**Systemic**	TSCI	47	50,865	0.92	1.92 (1.44 to 2.55)	1.73 (1.30 to 2.30)	1.50 to (1.13 to 2.00)
	Non-TSCI	53,838	1.4	0.39	1.00 reference	1.00 reference	1.00 reference

^A^Adjusted for age, calendar year and sex.

^B^Adjusted for age, calendar year, sex, and modified Charlson Comorbidity Index.

^C^Adjusted for age, calendar year, sex, modified Charlson Comorbidity Index, and hospitalization for (bacterial/viral/other) infections.

^D^Person years for non-traumatic spinal cord injury population presented in 10^8^ exponent.

^E^Study period from 1988-2018.

^F^Study population included from age of 12 years to let statistical model converge.

*PY* Person years, *TSCI* Traumatic spinal cord injury, *DM-1* Diabetes mellitus type 1, *MS* Multiple Sclerosis, *CCI* Charlson Comorbidity Index, *N* counts, *CI* Confidence interval.

For specification of autoimmune disease groups: See supplementary 2.

Table 3 shows the result for *all-incident*, *Gastroenterologic*, *DM-1*, *multiple sclerosis*, *Dermatologic*, and *Systemic* autoimmune diseases when TSCI was categorized as either cervical or thoracic. There were 130 cases of cervical TSCI and 106 cases of thoracic TSCI based on 29,512 and 21,192 person-years at risk, respectively. The *all-incident types* analysis suggests a tendency towards higher risk with thoracic TSCI for most outcomes, except for *Dermatologic* and *Systemic* types.

**Table 3:** Secondary study outcomes.

	Incidence rate ratios of autoimmune disease associated with traumatic spinal cord injury stratified by region of injury.						
					1: Basic adjustment^A^	2: CCI adjusted^B^	3: Fully adjusted^C^
Autoimmune disease group	Exposure status	N	PY^D^	Rate; 1000^-1^		Incidense rate ratio (95% CI)	
**All incident types**	c-TSCI	130	29,512	4.4	1.70 (1.43 to 2.02)	1.41 (1.18 to 1.67)	1.22 (1.03 to 1.45)
	t-TSCI	106	21,192	5.0	1.98 (1.63 to 2.39)	1.74 (1.44 to 2.10)	1.49 (1.23 to 1.80)
	Non-TSCI	274,314	1.4	2.0	1.00 reference	1.00 reference	1.00 reference
**Endocrine & haematologic**	..	..	..	..	..	..	..
**Gastroenterologic**	c-TSCI	20	29,512	0.68	1.15 (0.74 to 1.79)	0.97 (0.62 to 1.50)	0.84 (0.54 to 1.30)
	t-TSCI	22	21,192	1.0	1.82 (1.20 to 2.76)	1.62 (1.07 to 2.47)	1.40 (0.92 to 2.12)
	Non-TSCI	65,220	1.4	0.47	1.00 reference	1.00 reference	1.00 reference
**DM-1**^E^	c-TSCI	29	27,944	1.0	1.80 (1.25 to 2.59)	1.18 (0.82 to 1.69)	0.97 (0.67 to 1.39)
	t-TSCI	26	19,721	1.3	2.22 (1.58 to 3.42)	1.75 (1.19 to 2.57)	1.41 (0.96 to 2.07)
	Non-TSCI	51,846	1.2	0.45	1.00 reference	1.00 reference	1.00 reference
**Iridocyclitis**	..	..	..	..	..	..	..
**MS**^**F**^	c-TSCI	13	28,767	0.45	3.12 (1.81 to 5.38)	2.87 (1.67 to 4.95)	2.70 (1.56 to 4.65)
	t-TSCI	14	20,907	0.67	4.51 (2.67 to 7.62)	4.29 (2.54 to 7.25)	3.98 (2.36 to 6.72)
	Non-TSCI	15,709	1.1	0.15	1.00 reference	1.00 reference	1.00 reference
**Dermatologic**	c-TSCI	23	29,512	0.78	2.73 (1.81 to 4.11)	2.35 (1.56 to 3.54)	2.00 (1.33 to 3.00)
	t-TSCI	14	21,192	0.66	2.37 (1.40 to 4.00)	2.14 (1.27 to 3.61)	1.77 (1.05 to 3.00)
	Non-TSCI	28,164	1.4	0.20	1.00 reference	1.00 reference	1.00 reference
**Other neurologic**	..	..	..	..	..	..	..
**Systemic**	c-TSCI	29	29,512	0.98	2.01 (1.39 to 2.89)	1.79 (1.24 to 2.57)	1.57 (1.09 to 2.25)
	t-TSCI	18	21,192	0.85	1.81 (1.14 to 2.87)	1.66 (1.05 to 2.64)	1.43 (0.90 to 2.26)
	Non-TSCI	53,838	1.4	0.39	1.00 reference	1.00 reference	1.00 reference

^A^Adjusted for age, calendar year, and sex.

^B^Adjusted for age, calendar year, sex, and modified Charlson Comorbidity Index.

^C^Adjusted for age, calendar year, sex, modified Charlson Comorbidity Index, and hospitalization for (bacterial/viral/other) infections.

^D^Person years for non-traumatic spinal cord injury population presented in 10^8^ exponent.

^E^Study period 1988 – 2018.

^F^Study population included from age of 12 years to let statistical model converge.

*TSCI* Traumatic spinal cord injury, *N* Counts, *PY* Person years, *CI* Confidence interval, *c-TSCI* Cervical traumatic spinal cord injury, *t-TSCI* Thoracic traumatic spinal cord injury.

For specification of autoimmune disease group see Supplementary 2.

For specification of stratification of diagnoses in cervical and thoracic traumatic spinal cord injury see supplementary 1.

## Discussion

In this nationwide cohort study, we found that TSCI is associated with a higher IRR of autoimmune diseases, particularly those affecting the nervous system. Additionally, we provide an overview of the associations for various autoimmune diseases, grouped primarily by the affected organ.

Although the biological pathway is not fully understood, a possible biological sequence from TSCI to chronic neuroinflammation, leading to secondary neurologic injury, the activation of neuro-autoreactive lymphoid cells, and ultimately autoimmunity might explain our findings [4, 5, 7, 10, 13]. The strong association of neurologic autoimmune diseases with TSCI aligns with the biological effects observed in previous experimental and observational studies.

Following TSCI, the initial immunological response involves polymorph nuclear myeloid infiltration of the lesion site, followed by M1 and M2 macrophages within the first hours to days [5]. Subsequently T-cells, especially CD8+ T-cells, are present, peaking early and persisting at about 10% of the peak concentration [5, 10]. B-cell expansion, maturation, and ectopic lymphoid follicle formation occur in the injured spinal cord, producing autoreactive antibodies such as anti-myelin basic protein (anti-MBP) [6, 7]. This process resembles the pathology seen in multiple sclerosis[16]. Recent human TSCI studies quantify the immunological response aligning with earlier experimental data, particularly the non-resolving aspects of the inflammatory cascade that may lead to central nervous system autoimmunity [13, 14]. Our study underscores the role of anti-MBP in secondary morbidity among TSCI patients, highlights its association with multiple sclerosis and Idiopathic polyneuropathy, both characterized by a pathological demyelination [5, 6, 8, 14, 16].

The development of Systemic and Dermatologic autoimmune diseases might be supported by the findings of systemic anti-nuclear antibodies (ANA) and anti-DNA (dsDNA) antibodies following TSCI, similar to those seen in rheumatism, systemic lupus erythematosus and autoimmune dermatologic diseases [7, 8]. These studies, along with our findings, suggest a true biological pathway where the combined innate and adaptive immune response to TSCI can lead to the maturation and clonal expansion of autoreactive T- and B-cells, ultimately leading to autoimmune disease.

That the initial increased risk of *Gastroenterologic* and *DM-1* conditions, nullified in the Charlson comorbidity index and fully adjusted models, may indicate the influence of other factors on those associations. The impact of Charlson comorbidity index and hospitalization for infections, possibly arising from SCI-IDS and autonomic dysreflexia, emphasize their implications for multiorgan disease beyond autoimmune diseases.

SCI-IDS and inherited infectious susceptibility could mediate the pathway from TSCI to autoimmune disease [12]. Due to the small number of individuals with TSCI and an autoimmune disease, stratified analysis for formal mediation-analysis was not feasible [28]. Instead, we adjusted for *hospitalization for infection* to highlight its importance and the implicit risk, potentially modifiable pharmacologically. Interpretations should be cautious; we believe that only the direction, not the effect size, can be evaluated. The consistent direction of effect estimates suggests validity but does not rule out a potential collider bias.

We aimed to minimize misclassification of co-variates, suspecting that autoimmune diseases in the TSCI group could introduce detection bias or reversed causality, leading to differential misclassification. To mitigate this risk, we censored individuals diagnosed with TSCI and autoimmune disease during the same hospitalization, counting their risk time as non-TSCI, but excluding them as autoimmune disease cases. To avoid the confounding effects of multiple autoimmune diseases, we evaluated only an individual’s first autoimmune disease [19]. Despite these efforts, the multimorbidity and frequent hospitalizations of TSCI patients could lead to residual detection bias.

Table 3 suggests that thoracic TSCI predominantly increases the risk of autoimmune disease compared to a cervical TSCI. Initially, we hypothesized that cervical TSCI would be associated with a higher risk of autoimmune disease based on findings that higher injury levels amplify SCI-IDS and autonomic dysreflexia [3, 11]. Experimental studies have shown lesions above T9 reduce preganglionic inhibitory activity of postganglionic sympathetic nerves to the spleen, causing leukopenia and enhancing SCI-IDS [3, 11]. Similar findings apply to autonomic dysreflexia [3].

Our results indicate that non-penetrating thoracic TSCI might require more energy, leading to greater neurologic trauma and collateral damage, potentially explaining the higher risk of autoimmune disease despite less SCI-IDS and autonomic dysreflexia. Additionally, thoracic TSCI might result in more significant blood-spinal cord barrier disruption due to a higher probability of American Spinal Injury Association Impairment Scale (AIS) A injuries than cervical TSCI. Breakage of the blood-spinal cord barrier seems to be more prevalent and more pronounced in thoracic TSCI too [14]. Therefore, our findings could be more influenced by the magnitude of trauma than the level of TSCI.

Our definition of TSCI and the incidence (see Supplementary 5) aligns with Bjornshave Noe et al., report from the Spinal Cord Injury Centre of Western Denmark from 1990–2012 [29]. Our annual incidence data from 1981–2012 show realistic and relevant variation, but the increase from 2013-2018 might reflect misclassification. Table 1 shows the accumulated person years by calendar period. The TSCI population´s flattening curve and the steady state of the non-TSCI population in the study´s later period suggest possible bias from incidence issues in 2014–2018. Misclassified TSCIs may not have a higher autoimmune disease risk, potentially increasing type 2 error risk, but such bias cannot fully explain the association.

Our register-based study has limitations including small sample sizes in groups of TSCI patients with outcomes, and the inability to adjust for lifestyle factors like smoking, alcohol, body mass index, and familiar co-occurrence of autoimmune disorders. The positive predictive value of included variables is partially known and we cannot rule out misclassification from non-validated variables, though Danish registries are generally high quality [23]. The DM1 diagnosis has a high positive predictive value but unknown sensitivity, though a high sensitivity is expected given the clinical course [30].

The study population included all Danes born in Denmark, with complete public registration in the Danish Civil registration system, minimizing selection bias [22].

Several trauma-related factors including immediate treatment, pre-hospital vital parameters, pre-hospital transport time, initial AIS, decompressive surgery, and primary spinal cord tissue vitality could not be evaluated. These factors could illuminate the effect of secondary neurologic injury and inflammatory activity on autoimmune risk. The Danish TSCI demographics as described by Noe et al. [29] indicate that sharp and penetrating TSCI is rare, limiting our findings to blunt trauma TSCI populations.

Studying rare diseases risks large association deviations from small count variations. We advise the reader to consider raw counts, rates, and the full spectrum of estimated confidence intervals and associations. Based on our results, clinicians should monitor for potential late sequelae of TSCI. Prevention and medical optimization might reduce the Charlson comorbidity index´s impact and infection-related hospitalizations until further controlled studies are available.

## Conclusions

TSCI is associated with an increased risk of developing autoimmune diseases, particularly those affecting the nervous system, in an open nationwide cohort with long follow-up. Infections following TSCI may explain some of the risk of developing autoimmune diseases.

## Supplementary information

Supplementary

## Data Availability

The data that support the findings of this study are available from Statistics Denmark but restrictions apply to the availability of these data, which were used under license for the current study, and so are not publicly available. Data are however available from the authors upon reasonable request and with permission of Danish Data Protection Agency, Statistics Denmark, and the Danish Health Data Authority.
