# Supplementary material for: Traumatic spinal cord injury and its correlation to risk of autoimmune/-inflammatory disease
Source: Spinal Cord. 2024 Sep 11;62(11):642–50. doi: 10.1038/s41393-024-01026-0 (PMC11549038; doi:10.1038/s41393-024-01026-0)
Supplement: Supplementary file 1 — Supplementary [file 41393_2024_1026_MOESM1_ESM.docx]

supplementary

Table of contents

[Supplementary 1: Stratification of traumatic spinal cord injury in cervical and thoracic regions 2](#_Toc171628931)

[Supplementary 2: Diagnoses and related ICD-8 & -10 codes of traumatic spinal cord injury, autoimmune disease, spinal fracture, and cancer 3](#_Toc171628932)

[Supplementary 3: Charlson comorbidity index with associated disease groups, associated ICD-8 and -10 codes and applied modifications 5](#_Toc171628933)

[Supplementary 4: Definition of bacterial, viral and other infections. 6](#_Toc171628934)

[Supplementary 5: Incidence of traumatic spinal cord injury in the period 1977-2018. The studyperiod was 1980-2018. 8](#_Toc171628935)

[Supplementary 6: Incidence rate ratio of autoimmune disease grouped based on traumatic spinal cord injury diagnosis before 1995 or from or later than 1995. That newyear Denmark changed from ICD-8 to ICD-10 9](#_Toc171628936)

# Supplementary 1: Stratification of traumatic spinal cord injury in cervical and thoracic regions

| Stratification of traumatic spinal cord injury diagnoses | |  |  |  |
| --- | --- | --- | --- | --- |
| Level of injury | ICD-8 | ICD-8 conditioned | ICD-10 | ICD-10 conditioned |
| Cervical | 806.00, 806.10, 806.90, 958.00-958.10 | 958.03: Registered temporarily with on of: 805.00-805.09 | S140, S141X (minus 141A and 141C) | G82, G952: Registered temporarily with on of: S120-S127, S130, S131 |
| Thoracic | 806.21-806.33, 806.91, 806.92, 806.93, 958.11-958.19, 958.23, 958.31, 958.33. 958.91 | 958.13: Registered temporarily with on of: 805.19, 805.21, 805.23, 805.31, 805.33 | S240, S241 (minus S241A and D) | G82, G952: Registered temporarily with on of: S220, S221, S32, S230, S320, S231, S331 |
| Conditioned diagnoses shall be registered +/- 7 days from one of the added fracture diagnoses and must not be in relation with any cancer diagnisosis -10y/+7d from time of TSCI diagnosis. | | | | |

# Supplementary 2: Diagnoses and related ICD-8 & -10 codes of traumatic spinal cord injury, autoimmune disease, spinal fracture, and cancer

| Spinal cord injury | | |  |
| --- | --- | --- | --- |
| Disease | | ICD-8 | ICD-10 |
| Concussion and oedema of cervical spinal cord | | 958.00, 958.01, (958.03) | DS140 |
| Crush injury of cervical spinal cord | | 958.02, 958.04, 958.08, 958.09, 958.10 | DS141B, -D, -E |
| Concussion and oedema of thoracic spinal cord | | 958.11, 958.13, 958.19 | DS240 |
| Crush injury of thoracic spinal cord | | 958.12, 958.14, 958.18 | DS241B |
| Paraplegia and tetraplegia* | | 343.02, 343.03 | DG82 |
| Cord compression, unspecified* | | NA | DG952 |
|  | Autoimmune disease | |  |
| Group name | Disease | ICD-8 | ICD-10 |
| Endocrine & haematologic | Primary adrenocortical insufficiency | 255.10, 255.11, 255.12, 255.18, 255.19 | DE27.1 |
|  | Thyrotoxicosis | 242.00 | DE050 |
|  | Autoimmune thyroiditis | 245.03 | DE063 |
|  | Pernicious anemia | 281.00, 281.01, 281.08, 281.09 | DD51.0 |
|  | Autoimmune haemolytic anaemias | 283.90, 283.91 | DD591 |
|  | Immunogen thrombocytopenic purpura | 446.49 | DD693 |
| Gastroenterologic | Crohn disease | 563.01 | DK50 |
|  | Ulcerative colitis | 563.19 | DK51 |
|  | Celiac disease | 269.00 | DK900 |
|  | Autoimmune hepatitis | 571.93 | DK732 |
|  | Primary biliary cirrhosis | 571.90 | DK743 |
|  | Primary sclerosing cholangitis | 575.04 | DK830 |
| DM-1 | Type 1 diabetes | 249 | DE10 |
| Iridocyclitis | Iridocyclitis | 364 | DH20 |
| MS | Multiple sclerosis | 340 | DG35 |
| Dermatologic | Pemphigus | 694 except 694.05 | DL10 |
|  | Pemphigoid | 694.05 | DL12 |
|  | Alopecia areata | 704.00 | DL63 |
|  | Vitiligo | 709.01 | DL809 |
|  | Psoriasis vulgaris | 696.09, 696.10, 696.19 | DL40 except DL404 |
| Other neurologic | Idiopathic polyneuritis | 354 | DG610 |
|  | Myasthenia Gravis | 733.09 | DG700 |
| Systemic | Seropositive rheumatoid arthritis | 712.19, 712.39, 712.59 | DM05, DM06 |
|  | Juvenile rheumatoid arthritis | 712.09 | DM08 |
|  | Granulomatosis with polyangiitis | 446.29 | DM313 |
|  | Polymyalgia rheumatica | 446.30, 446.31, 446.39 | DM315, DM316, DM353 |
|  | Systemic lupus erythematosus with organ or system involvement | 734.19 | DM321, DM329 |
|  | Dermatopolymyositis | 716 | DM33 |
|  | Progressive systemic sclerosis (scleroderma) | 734.00, 734.01  734.02, 734.08  734.09 | DM34 |
|  | Other systemic involvement of connective tissue (Sjögren’s syndrome) | 734.90 | DM350 |
|  | Ankylosing spondylitis | 712.40 | DM459 |
| Spinal fracture | | |  |
| Disease | | ICD-8 | ICD-10 |
| Fracture of cervical spine | |  | DS120:DS127 |
| Fracture of thoracic spine | |  | DS220 |
| Multiple fractures of thoracic spine | |  | DS221 |
| Fracture of 1^st^ lumbar vertebrae | |  | DS320A |
| Traumatic cervical disc rupture | |  | DS130 |
| Traumatic thoracic disc rupture | |  | DS230 |
| Traumatic lumbar disc rupture | |  | DS320 |
| Luksation of cervical vertebrae | |  | DS131 |
| Luksation of thoracic vertebrae | |  | DS231 |
| Luksation of lumbar vertebrae | |  | DS331 |
| Cancer | | |  |
| Disease | | ICD-8 | ICD-10 |
| Any cancer** | | 14-20, | DC00:DC97 |
| *If a concomittant fracture diagnosis is present +/- 7days and no cancer diagnosis is present -10y/+7d from onset of diagnosis. **ICD-8: 225.31, 225.39, 225.49, 238.49, 238.59 and ICD-10 C720; cancer in spinal cord. Those patients were excluded. | | | |

# Supplementary 3: Charlson comorbidity index with associated disease groups, associated ICD-8 and -10 codes and applied modifications

| Charlson comorbidity index | |  |  |  |
| --- | --- | --- | --- | --- |
| Comorbidity group | ICD-8 | ICD-8 exclusions | ICD-10 | ICD-10 exclusions |
| Acute myocardial infarction | 410 | - | I21, I22, I23 | - |
| Heart failure | 427.09, 427.10, 427.11, 427.19, 428,99, 782.49 | - | I50, I11.0, I13.0, I13.2 | - |
| Peripheral vascular disease | 440, 441, 442, 443, 444, 445 | - | I70-I74, I77 | - |
| Cerebral vascular accident | 430-438 | - | I60-I69, G45, G46 | - |
| Dementia | 290.09-290.19, 293.09 | - | F00-F03, F051, G30 | - |
| Chronic pulmonary disease | 490-493, 515-518 | - | J40-J47, J60-67, J68.4, J70.1, J70.3, J84.1, J92.0, J96.1, J98.2, J98.3 | - |
| Connective tissue disorder | Excluded | 712, 716, 734, 446, 135.99 | Excluded | M05, M06, M08, M09, M30, M31, M32, M33, M34, M35, M36, D86 |
| Peptic ulcer | 530.91, 530.98, 531-534 | - | K22.1, K25-K28 |  |
| Mild liver disease | 571.09-571.89, 571.91-571.92, 571.94-571.99, 573.01, 573.04 | 571.90, 571.93 | K70.0-K70.3, K70.9, K71, K740-742, K744-K746, K76.0 | B18, K73, K743 |
| Diabetes | Excluded | 249.00, 249.06, 249.07, 249.09, 250.00, 250.06, 250.07, 250.09 | Excluded | E10.0, E10.1, E10.9, E11.0, E11.1, E11.9 |
| Hemiplegia | 344 | - | G81 | G82 |
| Moderate/severe liver disease | 403, 404, 580-583, 584, 590.09, 593.19, 753.10-753.19, 792 | - | I12, I13, N00-N05, N07, N11, N14, N17-N19, Q61 | - |
| Diabetes complication | 249.01-249.05, 249.08, 250-01-250.05, 250.08 | - | E10.2-E10.8, E11.2-E11.8 | - |
| Any tumor | 140-194 | - | C00-C75 | - |
| Leukemia | 204-207 | - | C91-C95 | - |
| Lymphoma | 200-203, 275.59 | - | C81-C85, C88, C90, C96 | - |
| Moderate/severe liver disease | 070.00, 070.02, 070.04, 070.06, 070.08, 456.00-456.09, 573.00 | - | B15.0, B16.0, B16.2, B19.0, K70.4, K72, K76.6, I85 | - |
| Metastatic solid cancer | 195-198, 199 | - | C76-C80 | - |
| AIDS | Excluded | 079.83 | Excluded | B21-B24 |
| AIDS: Acquired Immunodeficiency syndrome | | | | |

# Supplementary 4: Definition of bacterial, viral and other infections.

| Infection category | ICD-8 Codes | ICD-10 Codes |
| --- | --- | --- |
| Bacterial infection | 00009-00599,00809-00839,01099-01299, 013, 094, 01400-01899, 02009-02700,02701, 02708,03599, 03609,03610, 03999, 07399, 07699, 07984, 07939, 08899, 08900, 09049, 28940, 32009-32080, 322, 36202, 36600, 36700, 36800, 36805, 36900, 36901,  08099-08399, 09009, 09039, 09059-09399, 09500-09799, 09800-09929, 10009-10499, 36100-36101, 36108-36109, 10009-10499, 38000-38001, 38209-38299, 38309-38399, 39099-39199, 392, 42100-42109,  46100-46109, 10009-10499, 42000, 46201, 46300, 46403, 50199, 50300, 50302, 51000, 51008, 51009, 48101-48308, 50800-50803, 51300-51309, 52259, 52649, 52722, 52838, 52839,52903, 54001, 56200-56219,  56600-56702, 56708, 57703, 59700, 59703, 59000, 59901, 59906, 59009-59099,  59500-59502, 60100, 60102, 60400, 60401, 60739, 61101, 61100, 61200-61499,  61600-61603, 62009-62099, 62200-62219,  63000-63139, 63500-63599, 62949, 64000, 64002, 64009, 64010, 64012, 64020, 64029, 64090, 64092, 64100, 64102, 64110, 64112, 64120, 64122, 64130, 64132, 64140, 64142, 64150, 64152, 64160, 64162, 64170, 64172, 64190, 64192, 64209, 64229, 64300, 64302, 64380, 64382, 64390, 64392, 64490, 64492, 64500, 64502, 64510, 64512, 64520, 64522, 64530, 64532, 64540, 64542, 64550, 64552, 64560, 64562, 64570, 64572, 64580, 64582, 64590, 64592, 67000-67009, 68009-68099,  68108-68299, 68408-68409, 67801, 68501, 68399, 72031, 73299, 76109, 76309,  68600-68608, 71000-71009, 72000-72029 | A022C, A03-A05, A15-A22, A229, A229A, A229B, A229C, A229D, A23-A58, A65-A69, A7, B088D,E060A, E236A, D733,E321, G00, G02 G042, G042A, G050, G060-G062, G079, H000, H030, H043, H440, H050-051, H061, H601, H031A, H031C, H031E, H038A, H061A, H131A, H131H, H131L, H131N, H190A, H190C, H192C,H192E, H192G, H192H, H220D, H220F-G, H320E, H620A, H624A, H670A-B, H660-H664, H700,-701, H750C, H940A,  I301A-D, I320A-D, I00-I02, I398D-H, I410A-E, I430C, I520A, J01, J020, J030, J13-J15, J160, J170, J200-J202, J340, J340A,-D, J340I, J340J, J36, J383B-D, J387-G, J390, J391, J851-J853, J86, J860, J869 , J950A, J398A, J40, J399, J409, K046, K102, K040A, K046A, K052A, K102A-D, K102G-H, K112A, K130A, K140A, K209A, K113, K122, K351, K570--K579, K61, K628N, K650N, K67, K630, K670-K673, K810, K810A, K810C, K859A, K930, L00-L03, L030, L030H-J, L031-L039, L040-L089, M00, M010-M013, M015B, M016, M463, M490, M491, M492, M600, M650, M630A-B, M680A-F, M710, M730-731, M725A, M86, M900, M901A, M902A-902E, N10-N12, N136A-E, N151, N160A-B, N160E-F, N200I, N201I, N30, N300-N303, N303A, N308, N308A, N309, N330, N34, N340-341, N342B, N370A, N390, N410-N413, N431, N450, N459, N481-482, N490-N492, N498A, N498B, N499A-C, N510A-C, N512A, N512B, N511, N511E-J, N518C, N61, N70-N76, N760-N768, N768A, N768B, N770B, N740-N744, N764, N980, O030, O040, O070, O075, O080, O23, O035A-B, O045, O045A-B, O088D-F, O411, O411A-D, O753. O753A, O85, O86, O91, O910-911, O980-O982, O986-O989, P231-P236, P36, P360-P370, P38-39, P390, T814, T814A-D, T814F, T814G-J, T793, T802, T826-827,  T835-836, T845-847, T857, T874, T880, Y410, Y419, Z220-Z224, Z228-Z229 |
| Viral infection | 00880-00890, 040-044, 04509-04699,  05009-05200, 05201,05208-05301, 05302, 05303-05402, 05403, 05404-05500, 05501,  05508-05600, 05601, 05608-05799,  06009-06199, 06209-06599, 06709-06899, 07000-07009, 07199, 07200-07201, 07202, 07203-07209, 07409-07500, 07501,  07508-07509, 07809-07919, 07929,  07949-07982, 07983, 07989-07999, 09990, 46099, 46400-46402, 46408-46599,  47099-47309, 47400-47409, 48099, 76129, 76139 | A08, A080-A084, A60, A630, A80-A90, A9, B00, B000-B004, B004A, B005, B060A-C, B007-B009, B01, B010, B011, B011A, B012, B018-019, B02, B020-B023,  B027-028, B030, B03-B05, B050-054, B058, B06, B060, B068, B069, B07, B079, B08, B080, B081-B083,  O084-085, B088, B088A-B088C, B09, B15-B26,  B260-B263, B268, B269, B27, B270-271,B278-279, B30, B300-303, B308, B309, B33, B330-333, B338, B34,  B340-344, B348, B349, G020, G051, H031B, H031D, H031F, H131C, H131J, H131M, H190D, H19, H191B, H192B, H192D, H192I, H192J, H220C, H220E, H320, H320C, H621A-B, H622A-C, H671A, H671B, I400B, I411A, I411B, J00, J04-J06, J10-J12, J050, J171A-D, J203-207, J210, K770A, K770B, K871A, K871B, M015, M015A, M014, N518B, N770D, N771B, N771G, N771L, O353, O984-985, P230, P35, Z225-226 |
| Other types of infection | 00609-00799,00899,00999, 08409, 08419,08799,08990,09991-09999,  11000-11799, 13009,13019,13099, 13100-13609, 32089-32099, 32300, 36000, 38002-38009,38100-38101, 38108-38199,38400-38401, 42001-42009, 42199, 42299, 46200, 46208, 46209,  46301-46309, 46600-46601, 48100,  48309-48699, 50300-50306, 50308, 50309, 52720, 52809, 54000, 57201, 57209, 76149, 99859, 99939, 54008-54099,  68692-68694, 76319-76399 | A06, A060, A061-A066, A068-069, A07, A070-073,  A078-079, A085, A09, A099, A59, A63, A638, A64, A649, B375, B451, B50-60, B99, B64, B649, B85-B89, G02, G021A, G021B, G021C, G028A, G040, G049, G049A, G049B-C, G052A-C, G052E-G, G052H-J, G079D, H100, H102-H105, H108-109, H131O, H162, H162A, H192A, H192F, H441, H441A, H163, H169, H603, H628, H320D, H650-651, H669, H750, H750A-B, H940, H940B-C, I301, I301E, I400, I400A, I411-412, I412A-B, I521C, I300,  I308,-309, I33, J18, J028, J02-J03, J038-039, J172, J173A-C, J178, J20-22, J208-209, J218-219, J229, J32, J329, J350, J37, J370-371, J40-J42, J998B, J998C, K351A, K770C, K770D, K770E, M016C, M631D-F, M632A, P390B, K35, K350, K750, L303, M631C, M645, M651, M711, N160D, O358B, O983, O986-O989, P238-239, P369, P37, P371-375, P378-379, P390-P399, T89, Y4139, Z22 |

# Supplementary 5: Incidence of traumatic spinal cord injury in the period 1977-2018. The studyperiod was 1980-2018.

| **1976** | **1977** | **1978** | **1979** | **1980** | **1981** | **1982** | **1983** | **1984** | **1985** | **1986** | **1987** | **1988** | **1989** | **1990** |
| --- | --- | --- | --- | --- | --- | --- | --- | --- | --- | --- | --- | --- | --- | --- |
| \| 8 \| \| --- \| | \| 69 \| \| --- \| | \| 42 \| \| --- \| | \| 33 \| \| --- \| | \| 38 \| \| --- \| | \| 66 \| \| --- \| | \| 66 \| \| --- \| | \| 57 \| \| --- \| | \| 65 \| \| --- \| | \| 64 \| \| --- \| | \| 83 \| \| --- \| | \| 52 \| \| --- \| | \| 79 \| \| --- \| | \| 80 \| \| --- \| | \| 57 \| \| --- \| |
| **1991** | **1992** | **1993** | **1994** | **1995** | **1996** | **1997** | **1998** | **1999** | **2000** | **2001** | **2002** | **2003** | **2004** | **2005** |
| \| 85 \| \| --- \| | \| 73 \| \| --- \| | \| 73 \| \| --- \| | \| 77 \| \| --- \| | \| 80 \| \| --- \| | \| 72 \| \| --- \| | \| 65 \| \| --- \| | \| 57 \| \| --- \| | \| 66 \| \| --- \| | \| 97 \| \| --- \| | \| 65 \| \| --- \| | \| 76 \| \| --- \| | \| 68 \| \| --- \| | \| 59 \| \| --- \| | \| 74 \| \| --- \| |
| **2006** | **2007** | **2008** | **2009** | **2010** | **2011** | **2012** | **2013** | **2014** | **2015** | **2016** | **2017** | **2018** | **Total** |  |
| \| 69 \| \| --- \| | \| 64 \| \| --- \| | \| 77 \| \| --- \| | \| 77 \| \| --- \| | \| 78 \| \| --- \| | \| 65 \| \| --- \| | \| 87 \| \| --- \| | \| 98 \| \| --- \| | \| 112 \| \| --- \| | \| 113 \| \| --- \| | \| 118 \| \| --- \| | \| 168 \| \| --- \| | \| 200 \| \| --- \| | \| 3272 \| \| --- \| |  |

# Supplementary 6: Incidence rate ratio of autoimmune disease grouped based on traumatic spinal cord injury diagnosis before 1995 or from or later than 1995. That newyear Denmark changed from ICD-8 to ICD-10

Before 1995:

|  | **1: Basic adjust** | **2: CCI adjusted** | **3: Fully adjusted** |
| --- | --- | --- | --- |
| **Exposure status** |  | **IRR (95% CI)** |  |
| TSCI | 2.09 ( 1.42, 3.06) | 1.63 ( 1.11, 2.39) | 1.27 ( 0.86, 1.86) |
| Not | 1.00 reference | 1.00 reference | 1.00 reference |

From 1995 and later:

|  | **1: Basic adjust** | **2: CCI adjusted** | **3: Fully adjusted** |
| --- | --- | --- | --- |
| **Exposure status** |  | **IRR (95% CI)** |  |
| TSCI | 1.79 ( 1.56, 2.05) | 1.52 ( 1.33, 1.74) | 1.33 ( 1.16, 1.52) |
| Not | 1.00 reference | 1.00 reference | 1.00 reference |
